# Fatigue and Muscle Strength Involving Walking Speed in Parkinson's Disease: Insights for Developing Rehabilitation Strategy for PD

**DOI:** 10.1155/2017/1941980

**Published:** 2017-02-22

**Authors:** Ying-Zu Huang, Fang-Yu Chang, Wei-Chia Liu, Yu-Fen Chuang, Li-Ling Chuang, Ya-Ju Chang

**Affiliations:** ^1^Department of Neurology, Chang Gung Memorial Hospital, Linkou Medical Center, 5 Fusing St., Kweishan, Taoyuan 333, Taiwan; ^2^School of Medicine, College of Medicine, Chang Gung University, 259 Wen-Hwa 1st Rd, Kweishan, Taoyuan 333, Taiwan; ^3^Neuroscience Research Center, Chang Gung Memorial Hospital, Linkou Medical Center, 5 Fusing St., Kweishan, Taoyuan 333, Taiwan; ^4^Institute of Cognitive Neuroscience, National Central University, 300 Zhongda Rd., Zhongli, Taoyuan 320, Taiwan; ^5^Department of Neurology, School of Medicine, Fukushima Medical University, 1 Hikarigaoka, Fukushima 960-1295, Japan; ^6^Department of Physical Therapy and Graduate Institute of Rehabilitation Science, College of Medicine and Healthy Aging Research Center, Chang Gung University, 259 Wen-Hwa 1st Rd, Kweishan, Taoyuan 333, Taiwan; ^7^Department of Physical Medicine and Rehabilitation, Chang Gung Memorial Hospital, Linkou Medical Center, 5 Fusing St., Kweishan, Taoyuan 333, Taiwan

## Abstract

*Background*. Problems with gait in Parkinson's disease (PD) are a challenge in neurorehabilitation, partly because the mechanisms causing the walking disability are unclear. Weakness and fatigue, which may significantly influence gait, are commonly reported by patients with PD. Hence, the aim of this study was to investigate the association between weakness and fatigue and walking ability in patients with PD.* Methods*. We recruited 25 patients with idiopathic PD and 25 age-matched healthy adults. The maximum voluntary contraction (MVC), twitch force, and voluntary activation levels were measured before and after a knee fatigue exercise. General fatigue, central fatigue, and peripheral fatigue were quantified by exercise-induced changes in MVC, twitch force, and activation level. In addition, subjective fatigue was measured using the Multidimensional Fatigue Inventory (MFI) and Fatigue Severity Scale (FSS).* Results*. The patients with PD had lower activation levels, more central fatigue, and more subjective fatigue than the healthy controls. There were no significant differences in twitch force or peripheral fatigue index between the two groups. The reduction in walking speed was related to the loss of peripheral strength and PD itself.* Conclusion*. Fatigue and weakness of central origin were related to PD, while peripheral strength was important for walking ability. The results suggest that rehabilitation programs for PD should focus on improving both central and peripheral components of force.

## 1. Introduction

Gait disturbance significantly affects the quality of life in patients with Parkinson's disease (PD), particularly in the later stages. Due to the failure of current drug treatment for gait problems in patients with PD, neurorehabilitation programs are gaining popularity. However, such problems are also a big challenge for neurorehabilitation because the mechanisms causing the walking disability in PD are largely unknown. Fatigue and weakness are prominent symptoms in most PD patients, and both can affect the life quality and functional walking ability [1, 2]. Weakness of the lower extremities has been reported to be a risk factor for indoor falls in patients with PD [3]. Fatigue that occurs at the early stage and then progresses as the disease advances affects about half of the patients with idiopathic PD [2, 4], and PD-related weakness and fatigue have been linked to the severity and duration of PD, levodopa dose, activation failure, and comorbidities such as depression and anxiety [4–6]. Chou and colleagues [7] reported that deep brain stimulation surgery did not change levels of PD-related fatigue. Understanding the mechanisms causing weakness and fatigue would be beneficial in developing suitable rehabilitation strategies for patients with PD.

Fatigue is a complicated disorder that has several domains, including physical fatigue, mental fatigue, reduced activity, and reduced motivation [8]. Recent studies have suggested that PD-related fatigue is both a nonmotor and a motor symptom [9, 10]. Fatigue in PD is commonly evaluated by questionnaire-based scales such as the Fatigue Severity Scale (FSS) [11] and Multidimensional Fatigue Inventory (MFI) [8, 12]. For example, Lou and colleagues found that PD patients suffered more fatigue than healthy controls in mental and physical domains using the MFI [12]. These questionnaire-based fatigue scales are convenient for screening fatigue; however they are subjective and cannot identify the cause or mechanism of fatigue.

The mechanisms of PD-related weakness and fatigue have yet to be clarified. Central nervous system- (CNS-) related factors (central fatigue) and peripheral factors (peripheral fatigue) may both contribute to weakness and fatigue [13]. It has been shown that fatigue in patients with CNS disorders such as multiple sclerosis involve both central and peripheral components. Central fatigue may include mental fatigue and a decrease in motivation [14, 15]. In contrast, peripheral fatigue may result from neuromuscular transmission failure along *α* motor neurons, neuromuscular junctions, muscle cell membranes, and factors within muscle fibers such as E-C coupling failure [16]. Recent studies have reported that the activation level (VA) of the maximum voluntary muscle contraction (VA) is lower in patients with PD than in age-matched controls [4, 17]. This suggests that PD patients are prone to have central fatigue; however this phenomenon has never been quantified.

In the laboratory, the total amount of fatigue can be quantified by the fatigue index, which is the ratio of maximum voluntary contraction (MVC) force before versus after fatigue-inducing exercise. Peripheral fatigue is commonly measured by the decrease in a muscle twitch force elicited by electrical stimulation of the peripheral nerve [18], while central fatigue is commonly quantified as the decrease in VA after fatigue-inducing exercise [13]. Quantifying the degree of central versus peripheral fatigue in PD patients is important for the development of suitable drug and rehabilitation interventions. Therefore, the aims of this study were to (1) investigate the level and mechanism of lower limb weakness and fatigue and (2) correlate the measured components to walking speed in patients with idiopathic PD.

## 2. Materials and Methods

### 2.1. Participants

The PD group included 25 patients (21 males, 4 females, mean age: 62.12 ± 10.23 years) with idiopathic PD recruited from the outpatient clinics at the Linkou Branch of Chang Gung Memorial Hospital in Taiwan (Table 1). Twenty-five healthy adults (8 males, 17 females, mean age: 59.04 ± 9.13 years) were recruited from the community as the healthy control (HC) group. The inclusion criteria for the PD group were (1) PD diagnosed according to the United Kingdom Brain Bank Criteria, (2) with Hoehn and Yahr stages II-III, (3) stable medication usage, and (4) Mini-Mental State Examination score ≥ 24. All PD patients were tested during a clinical “ON” status, with the more severe side being tested. The patients who had tremors when on medication or during recording and those with other central or peripheral neurological diseases or musculoskeletal injuries of the lower limbs were excluded from the study. Only the subjects with a sedentary lifestyle without regular exercise were recruited in both groups to avoid the confounding factor of physical activity level. Written informed consent was obtained from all subjects before participation. This study was approved by the Chang Gung Medical Foundation Institutional Review Board.

### 2.2. Evaluation of Subjective Fatigue

Subjective fatigue was evaluated in all subjects using the FSS, a 9-item statement rating the severity of fatigue, and the MFI, a 20-item self-report instrument designed to measure fatigue. Both tools have been reported to have good validity and reliability [8], and both were carefully explained by an examiner who was blind to the purpose of this study.

### 2.3. Experimental Design

After a 30-minute rest, the force of MVC, VA level, twitch force, and fatigue indexes were evaluated. The subjects were seated on a custom-made knee extension force measurement system, which included a force transducer (AWU, Genisco Technology, CA, USA) coupled to a transducer amplifier (Gould Inc., Valley View, OH, USA), to measure the knee isometric extension force at 90 degrees of flexion [14]. Responses were sampled at 1000 Hz and recorded on a computer using a Power 1401 laboratory interface (Cambridge Electronic Design, Cambridge, UK) for offline analysis.

### 2.4. Maximum Voluntary Contraction (MVC)

To record the MVC of the quadriceps muscle, each subject performed three MVCs to warm up, followed by five MVCs which were recorded. The force trace was displayed on an oscilloscope (MetraByte AS 1600, Keithley Instruments, Inc., Cleveland, OH, USA) for real-time feedback. When performing MVC, the subjects were instructed to fully contract the quadriceps muscle for 5 seconds. Both verbal encouragement and visual feedback were given during the contraction. A rest period of 10 seconds was given between consecutive contractions. The amplitude of the MVC force was calculated from the force-time curve. To avoid possible changes in force contraction velocity before and after the fatigue-inducing exercise, the amplitude of MVC force was calculated by averaging the force level from the force peak until 0.5 s after the peak in each MVC.

### 2.5. Voluntary Activation Level (VA) Test and Twitch Forces

VA was measured using the interpolated twitch test [14, 19]. During the test, the quadriceps muscle was stimulated (Digitimer DS7A, Digitimer Ltd., Welwyn Court, UK) with surface electrodes. The pulse width of stimulation was 200 *μ*s, and the stimulation intensity was supramaximal, that is, 120% of the intensity eliciting the maximum resting twitch. The supramaximal stimulus was delivered when the quadriceps was at rest and during MVC to elicit the resting twitch and the interpolated twitch (*T*2), respectively. The resting twitches were measured before and after MVC to obtain unpotentiated and potentiated resting twitches, respectively. Only the potentiated resting twitches were used (*T*1). The twitch forces were measured as the peak amplitude of the twitches, and VA was calculated using the following formula:

(1) $$\begin{array}{c} VA=\left(\;1-\frac{T2}{T1}\;\right)\times 100\%. \end{array}$$

**Figure 1:**
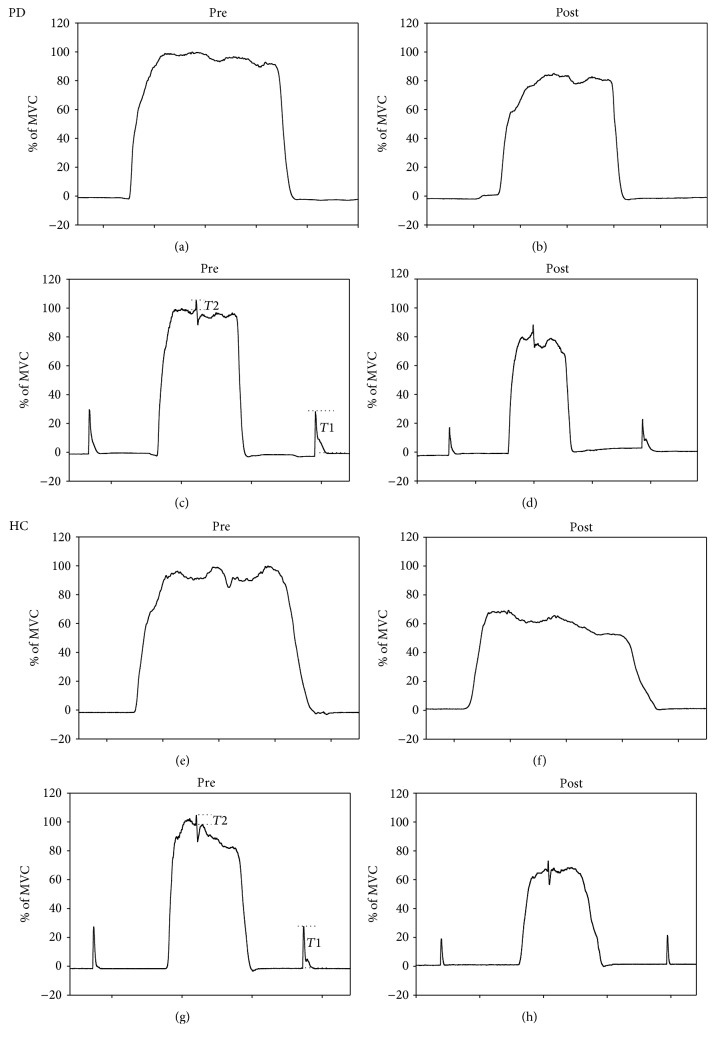
Representative force-time curves of MVC, twitch force, and interpolated twitch for one PD patient ((a)–(d)) and one healthy subject ((e)–(h)) before (pre) and after (post) fatigue. The *y*-axis shows the percentage of the peak MVC, with the prefatigue maximum set to 100%. *T*_1_: potentiated twitch was also used to represent twitch force.

The subjects then underwent the fatigue task, in which they were asked to repeat 5-second isometric MVCs of the quadriceps muscle, with 10-second rest periods in between, for 15 minutes. The subjects were encouraged verbally and visual feedback was provided to increase motivation during MVC. The MVC, VA, and twitch force were determined again after the fatigue task. Representative data for MVC, twitch force, and interpolated twitch force are shown in Figure 1(c).

### 2.6. Fatigue Indexes

The general fatigue index (GFI) was calculated as the ratio of postfatigue MVC to prefatigue MVC, and the central fatigue index (CFI) was calculated as the ratio of postfatigue VA to prefatigue VA. Central fatigue refers to a progressive decline in the ability to activate muscles voluntarily, and it has been attributed to impairment at sites of suprasegmental structures [13, 20]. By calculating the change in VA caused by exercise, exercise-induced central fatigue can be quantified. The peripheral fatigue index (PFI) was calculated as the ratio of the postfatigue twitch force to the prefatigue twitch force [14, 15]. The GFI, CFI, and PFI had values between 0 and 1, with a higher value indicating less general fatigue, central fatigue, and peripheral fatigue, respectively.

### 2.7. Walking Test

Functional ambulation ability was evaluated using a 6.5 m walking test. The subjects were asked to walk 6.5 m without assistance. To eliminate the influence of acceleration and deceleration, the average walking speed was measured over the middle 4.5 m.

### 2.8. Data Analysis

One-way ANOVA was used to analyze between-group differences in MVC, VA, twitch force, GFI, CFI, PFI, MFI, FSS, and walking speed (version 9.2, SAS Institute, Cary, NC, USA). Spearman correlation was used to analyze the correlations among levodopa equivalent dose (LED), UPDRS part III (motor part), and different components of fatigue. Stepwise regression analysis was used to identify the factors contributing to walking speed. The significance level was set at *p* < 0.05.

## 3. Results

All demographic and clinical data are shown in Table 1. There was no significant difference in age between the PD (62.12 ± 10.23 years) and HC (59.04 ± 9.13 years) groups (*p* = 0.49). The average LED of the patients was 258.92 ± 104.30 (range: 100–500) mg/day.

**Figure 2:**
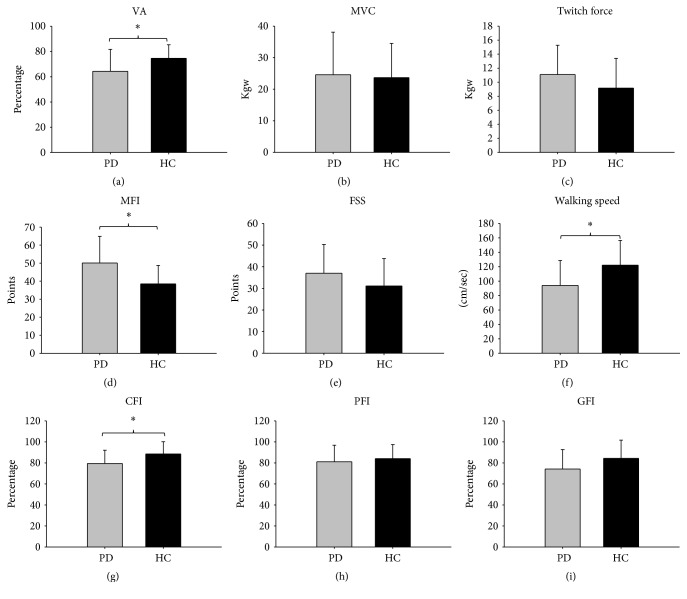
Differences between the PD and HC groups in (a) VA, (b) MVC, (c) twitch force, (d) MFI, (e) FSS, (f) walking speed test, exercise-induced (g) central fatigue, (h) peripheral fatigue, and (i) general fatigue indexes in the PD and HC groups. ^*∗*^*p* < 0.05.

Figure 1 shows the representative force-time curves of MVC, twitch force, and interpolated twitch for one PD patient ((a)–(d)) and one healthy subject ((e)–(h)) before (pre) and after (post) fatigue tests. Between-group comparisons are shown in Figure 2. In the prefatigue state, VA was lower in the PD group (64.35 ± 17.37%) than in the HC group (74.65 ± 10.71%) (*F*(1, 48) = 6.36, *p* = 0.02; Figure 2(a)), suggesting that weakness originated from central fatigue in the PD group. There were no significant differences in MVC (*F*(1, 48) = 0.07, *p* = 0.79) or twitch force (*F*(1, 48) = 2.64, *p* = 0.11) (Figures 2(b) and 2(c)) between the two groups. The PD group had more subjective fatigue (MFI = 50.08 ± 14.80) than the control group (MFI = 38.52 ± 10.22) (*F*(1, 48) = 10.33, *p* < 0.01, Figure 2(d)), while no significant difference was found in FSS between the two groups (PD = 37 ± 13.28, control = 31.08 ± 12.67, *F*(1, 48) = 2.6, *p* = 0.11, Figure 2(e)). The PD patients had a slower walking speed (93.99 ± 34.6 cm/sec) than the HC group (122.17 ± 34.27 cm/sec) (*F*(1, 47) = 10.93, *p* < 0.01, Figure 2(f)). With regard to the fatigue indexes, fatigue-inducing exercise was associated with a significantly lower CFI in the PD group (79.48 ± 12.67) than in the HC group (88.53 ± 11.68) (*F*(1, 48) = 6.9, *p* = 0.01), suggesting that the PD patients experienced fatigue of central origin more easily than the healthy subjects (Figure 2(g)). No between-group difference was observed in PFI (PD: PFI = 81.13 ± 15.71, HC PFI = 84.15 ± 13.37, *F*(1, 48) = 0.54, and *p* = 0.47), suggesting that both groups had similar levels of peripheral fatigue (Figure 2(h)). GFI was marginally lower in the PD group (74.22 ± 18.52) than in the HC group (84.37 ± 17.31%) (*F*(1, 48) = 4, *p* = 0.05, Figure 2(i)).

We further performed correlation analysis between measures (Table 2). Pearson correlation coefficients showed that MVC correlated with both VA (*r* = 0.56, *p* < 0.001) and resting twitch (*r* = 0.74, *p* < 0.001) in the PD group, whereas it correlated only with twitch force (*r* = 0.71, *p* < 0.001) in the HC group. No significant correlations were found between MFI and objective fatigue parameters including GFI (PD: *p* = 0.27, HC: *p* = 0.92), CFI (PD: *p* = 0.25, HC: *p* = 0.42), or PFI (PD: *p* = 0.52, HC: *p* = 0.76). Stepwise regression analysis revealed that walking speed could be affected by having PD itself and by twitch force (*R*-square = 0.23, *p* < 0.01).

The spearman correlation analysis showed that LED and VA correlated with the UPDRS III score (*r* = −0.63, *p* = 0.022 and *r* = −0.65, *p* = 0.025, resp.), but not with other parts. Moreover, we did not find any correlations between LED and different types of fatigue (GFI: *r* = 0.12, *p* = 0.57; CFI: *r* = 0.25, *p* = 0.22; and PFI: *r* = 0.20, *p* = 0.34) and/or between UPRDS UPDRS III and different types of fatigue (GFI: *r* = 0.10, *p* = 0.75; CFI: *r* = −0.41, *p* = 0.16; and PFI: *r* = 0.03, *p* = 0.92).

## 4. Discussion

The current study revealed that PD patients had lower VA, lower CFI, and more subjective fatigue than the HCs. The MVC of the PD group correlated with both VA and twitch force, whereas the MVC of the control group correlated only with the twitch force. The slower walking speed in the PD patients could be explained by both having the disease of PD and loss of twitch forces.

The finding of a lower VA in the patients with PD is consistent with previous studies [4, 17]. VA reflects the ability of the CNS to drive the muscular system without being confounded by peripheral muscle strength [19]. A lower VA suggests that PD patients have subclinical weakness of central origin. The lack of a significant difference in twitch force between the PD and HC groups further confirms that a peripheral mechanism may not be involved. In terms of fatigue, the PD patients had a lower CFI than the HCs, suggesting that the PD patients had more fatigue of central origin after exercise. Quantification of exercise-induced fatigue has seldom been studied in PD. Although a lower activation level and higher general fatigue have been reported [4, 17], central and peripheral exercise-induced fatigue have never been investigated separately in patients with PD. To the best of our knowledge, this is the first study to identify exercise-induced central and peripheral fatigue in PD patients using a well-established laboratory technique that has been used in other neurological diseases such as multiple sclerosis [14]. Stevens-Lapsley et al. reported that general fatigue in the quadriceps muscle was only greater in PD patients with a low motor score but not in those with a high motor score compared to controls [4]. This is consistent with our results which revealed only a marginally lower GFI in the PD patients. Together with the finding of no difference in PFI between the PD and HC groups, the current study confirms that fatigue in PD is of central origin and that only the CFI is sensitive enough to detect such fatigue.

In this study, the PD group had more exercise-induced central fatigue and reported a higher MFI compared to the HC group. However, no significant correlation was identified between the MFI and CFI. The scale of the MFI, a self-reported psychometric measurement instrument, is not linear [8], and this nonlinearity is probably the cause of the poor correlation with the CFI.

The MVC in the PD group was significantly correlated with both the forces of central (VA) and peripheral (resting twitch) origin, whereas the MVC in the HC group was only correlated with the force of peripheral origin. In addition, there was no difference in the twitch force between the two groups. It is generally accepted that age-related weakness is a result of peripheral muscle weakness rather than reduced neural drive. A study on the force of the quadriceps muscle reported an approximately 50% lower twitch force but no change in either mean motor unit firing rates or activation level in older compared to younger subjects [21]. The possible factors contributing to peripheral weakness include a reduction in dietary protein, humoral effects of gonadal steroids, increases in catabolic stimuli, and decreased levels of physical activity [22]. The correlation between MCV and VA in the PD patients and the similar twitch force between the PD and HC groups suggest that, in addition to peripheral weakness seen in the elderly, PD patients suffer from weakness of central origin.

No significant difference in MVC or GFI (calculated from the change in MVC) was found between the PD and HC groups, suggesting that MVC alone is not sensitive enough to identify PD-related weakness. MVC measures both the central and peripheral components of fatigue [19, 23], and it is possible that a significant change in the central component, for example, lower VA, in the patients with PD contributed much less than the peripheral component resulting in similar changes in the PD and HC groups to MVC. On the other hand, the lower VA could be compensated by the increase in variability of firing rate of single motor units in PD [24]. According to the force-frequency relationship, the MVC force could be influenced by motor unit firing characteristics even with all the motor units fully recruited by the CNS [25].

The underlying mechanisms of PD-related activation failure and central fatigue are complicated. Central fatigue represents a failure of physical and mental tasks that require self-motivation and internal cues in the absence of demonstrable cognitive failure or motor weakness. Serotonin and dopamine have been identified as critical neurotransmitters in fatigue [26]. An animal study showed that levels of extracellular dopamine and 5-HT neurotransmitters increased significantly during exhausting exercise [27]. A reduced level of dopamine has also been reported in fatigued rats [28]. The reuptake of dopamine, but not the regulation of serotonin levels, has been reported to restore performance to some extent and in particular heat-mediated central fatigue [28, 29]. Pharmacological evidence further supports the importance of dopamine in fatigue [28]. In addition to dopamine deficiency, several lines of evidence suggest that the serotonergic system is also involved in the pathophysiology of PD [30, 31]. The failure of activation and central fatigue in PD patients is likely due to aberrant dopamine and serotonin systems. The potential mechanism may be related to dopamine transporter binding in the posterior putamen, the functional organization of basal ganglia, and connections of the basal ganglia to cortical motor and premotor areas [6]. Basal ganglia are involved in the limbic modification of cortical motor output via the dopaminergic system and the serotonin pathway [32]. Such limbic modification could affect motivation, thereby influencing the ability to sustain voluntary activation after exercise. However, we failed to find a correlation between LED or UPDRS III and fatigue measures. This is perhaps not surprising, because the experiments were performed when the patients were still taking medications, and medications are likely to help partially compensate for fatigue. Further studies including patients not taking medications are warranted to evaluate the correlation between daily levodopa supplements and serotonin-related factors such as depression and fatigue measures.

Other mechanisms also contribute to central fatigue. For example, central fatigue may result from insufficient drive from supraspinal sites [13, 20] because of a lack of subject motivation [32]. Recent studies have shown that during exhaustive exercise, group III/IV muscle afferents inhibit the motor cortex and promote central fatigue [33, 34]. During exercise, inadequate oxygen delivery to the brain may contribute to the development of fatigue [28, 35]. Future studies should focus on whether PD patients are more sensitive to group III/V inhibition and more vulnerable to inadequate brain oxygen delivery.

We also found a reduced walking speed in the PD patients, with an average of 93.99 ± 34.6 m/s in the PD group compared to 122.17 ± 34.27 m/s in the HC group, consistent with the study by Yang et al. [33]. Furthermore, we found that this reduction in walking speed could be partially explained by the peripheral component of knee extensor force. Although the correlation was not enough to infer their causal relationship, this finding is compatible with a previous study which demonstrated that muscle power was a significant determinant of walking speed in patients with PD even after adjusting for UPDRS motor score [34]. Therefore, improvements in peripheral muscular strength may help to improve the walking ability of PD patients. However, it should be noted that several other factors such as postural abnormalities, shorter stride, smaller forward moment velocity, and abnormal trunk muscle strength may also slow the walking speed in patients with PD [33].

### 4.1. Limitations

Tremors that may potentiate resting twitch force may have been a confounding factor in this study. However, we excluded subjects with obvious tremors and those with tremors during recording to avoid this issue. Moreover, only potentiated twitches recorded during muscle activation were analyzed. Thus, the influence of tremors was minimized by the experimental design. Another potential limitation is that we did not balance the gender distribution in the two groups. The reported influence of gender on fatigue has been inconsistent, and a gender difference has been reported in muscles of the upper extremities [35], but not of the lower extremities [35, 36]. In the present study, muscles in the lower extremities, that is, the quadriceps muscles, were evaluated and no difference in the GFI of knee extensor was found between groups. Hence, the difference in gender distribution is unlikely to have influenced the results.

## 5. Conclusion

In this study, we found that PD patients suffered from weakness of central origin in the prefatigue state. The patients reported more subjective fatigue and presented with more exercise-induced central fatigue than the HCs. In addition, peripheral strength was found to be an important factor with regard to the walking ability of the patients with PD. These results provide an insight into the mechanism of weakness and gait problems and may help with the development of rehabilitation programs for patients with PD in improving activation level, overcoming central fatigue and subjective fatigue, which will in turn be helpful to overcome PD-related weakness and fatigue. Peripheral muscle strength should be enhanced to improve walking speed.

## Figures and Tables

**Table 1:** Characteristics of the study subjects.

Group	PD (*N* = 25)	HC (*N* = 25)
Gender (female/male)	4/21	17/8
Age (years)	62.12 ± 10.23	59.04 ± 9.13
Height (cm)	167.04 ± 8.51	159.26 ± 8.89
Weight (kg)	68.44 ± 11.58	59.83 ± 11.17
Modified Hoehn and Yahr (HY) score, *N*		
HY = 1	6	—
HY = 1.5	5	—
HY = 2	6	—
HY = 2.5	4	—
HY = 3	4	—

**Table 2:** Correlation analysis between variables in the two groups.

Correlation	PD						HC					
	MFI		MVC		Speed		MFI		MVC		Speed	
	*r*	*p*	*r*	*p*	*r*	*p*	*r*	*p*	*r*	*p*	*r*	*p*
CFI	−0.24	0.25					0.17	0.42				
GFI	−0.23	0.27					−0.02	0.92				
PFI	−0.13	0.52					−0.06	0.76				
VA			0.56	<0.01^*∗*^	−0.07	0.73			0.38	0.06	0	0.99
TW			0.74	<0.01^*∗*^	0.28	0.19			0.71	<0.01^*∗*^	0.18	0.40
MVC					0.16	0.45					−0.14	0.51

PD, Parkinson's disease; HC, healthy control; MFI, Multidimensional Fatigue Inventory; CFI, central fatigue index; PFI, peripheral fatigue index; GFI, general fatigue index; VA, activation level; TW, twitch force; MVC, maximum voluntary contraction.

^*^Significant correlation, *p* < .05.
